# Occupational Exposure to Mycotoxins—Different Sampling Strategies Telling a Common Story Regarding Occupational Studies Performed in Portugal (2012–2020)

**DOI:** 10.3390/toxins12080513

**Published:** 2020-08-11

**Authors:** Susana Viegas, Carla Viegas, Carla Martins, Ricardo Assunção

**Affiliations:** 1 NOVA National School of Public Health, Public Health Research Centre, Universidade NOVA de Lisboa, 1600-560 Lisboa, Portugal; carla.viegas@estesl.ipl.pt (C.V.); carla.martins@insa.min-saude.pt (C.M.); ricardo.assuncao@insa.min-saude.pt (R.A.); 2 Comprehensive Health Research Center (CHRC), NOVA Medical School, Faculty of Medical Sciences, Universidade NOVA de Lisboa, 1169-056 Lisboa, Portugal; 3 H&TRC—Health & Technology Research Center, ESTeSL—Escola Superior de Tecnologia da Saúde, Instituto Politécnico de Lisboa, 1990-096 Lisboa, Portugal; 4 Food and Nutrition Department, National Institute of Health Dr. Ricardo Jorge, 1600-560 Lisboa, Portugal; 5 CESAM, Centre for Environmental and Marine Studies, University of Aveiro, 3810-193 Aveiro, Portugal

**Keywords:** workplace environments, exposure assessment, sampling strategy, biomonitoring

## Abstract

In occupational settings where exposure to organic dust occurs (e.g., intensive animal production, waste management, farming and many others) workers can also be exposed to mycotoxins. However, recognizing exposure to mycotoxins in workplace environments does not happen commonly and, consequently, remains as a not identified occupational risk factor. In the last decade, work developed in different occupational settings, using different sampling approaches reported that occupational exposure to mycotoxins occurs and it’s of upmost importance to be seen as an occupational concern that needs to be tackled. This paper intends to discuss the several possibilities available for assessing and characterizing the occupational exposure to mycotoxins through the description of the advantages and limitations of the different sampling strategies. Overviewing the approaches and the main achievements used in several field campaigns developed in Portugal, the knowledge obtained will be used to support the identification of the main aspects to consider when designing new occupational studies. The need for additional research work will also be discussed where new directions to follow will be debated.

## 1. Exposure to Mycotoxins in Occupational Environments

Workers in several activity sectors are exposed to organic dust that contains different bacteria and fungi, as well as their components such as mycotoxins, endotoxins and glucans. High exposure to organic dust containing mycotoxins, secondary toxic metabolites produced by fungi, can occur during the development of several working routine activities, such as storage work, loading, handling, or milling contaminated materials (e.g., grain, waste, and feed) in different types of industries (e.g., brewing, bakeries), and others such as caring for animals in animal husbandry settings [1,2,3,4,5,6,7,8]. Despite this occupational exposure has been demonstrated in previous research work [9,10,11,12,13,14,15,16,17,18,19], exposure assessment is not routinely performed and mycotoxins are still not recognized as an occupational risk factor present in several workplaces [20].

The negative health effects associated with human exposure to mycotoxins have been already described for some mycotoxins when exposure occurs by ingestion of contaminated foods. However, when recognizing exposure to mycotoxins in workplace environments, different exposure routes should be considered, namely inhalation and dermal absorption. Nevertheless, the health effects following inhalation or dermal contact are insufficiently described [20].

Therefore, it is particularly relevant to properly characterize occupational exposure through the identification of the present mycotoxins, their levels, duration and main routes of exposure associated to the specific occupational environments to understand the main determinants that may have an impact in exposure. Additionally, standardized methodologies (sampling and analysis) are needed to permit comparisons between the different studies [20]. Furthermore, until now, there have been insufficient epidemiological studies to evaluate the acute and chronic health effects of occupational exposure to provide a detailed picture of the health risks associated. Although with several challenges associated since one mycotoxin can cause several effects at different exposure levels, this is also crucial to the future development of occupational exposure limits for single mycotoxins and for mixtures of mycotoxins that are associated to similar health effects or sharing the same mode of action [20,21].

Considering the above, this paper intends to discuss the several possibilities available for assessing and characterizing the occupational exposure to mycotoxins through the description of the advantages and limitations of the different sampling strategies. Overviewing the approaches and the main achievements used in several field campaigns developed in Portugal, the knowledge obtained will be used to support the identification of the main aspects to consider when designing new occupational studies. The need for additional research work will also be tackled where new directions to follow will be discussed.

## 2. Occupational Exposure Assessment—Approach to Follow

Exposures occurring in the workplaces are known to have several causes for variation among individual workers or groups of workers, such as job tasks differences (between-worker variance), way to perform those tasks and tasks that only occur in some days of the week or even in some shifts of the day. Depending on the exposure assessment objective, a carefully planned sampling strategy is desirable so that measurements can be collected efficiently, and resources spent wisely [22]. The sampling strategy adopted should give results allowing to draw conclusions about exposure (how, when and who), risk and the guidance for the most suitable risk management measures to put in place. For this, the EN 689:2020 (Workplace Exposure—Measurement of Exposure by Inhalation to Chemical Agents—Strategy for Testing Compliance with Occupational Exposure Limit Values) can be followed and adapted according to the specific objectives of the study. However, this EN mentions as a first step the need to identify the chemical agents present in the workplace based on safety data sheets and other information that do not lead to mycotoxins since these are not a raw material or a final product and are, instead, an undesirable contaminant of the raw materials or workplace environment. Therefore, recognize mycotoxins presence in the workplace is a challenging goal and requires specific expertise, with only an experimented occupational hygienist, being able to identify the possible presence of mycotoxins and define a suitable monitoring campaign, can achieve.

Another major aspect is the fact that occupational exposure data are often collected without adequate or sufficient contextual information about the workplace impairing the effective use of data for exposure assessment purposes [20,23]. Therefore, the importance of collecting contextual information that allows understanding and identifying the exposure sources and determinants of exposure is of high relevance and can significantly improve our understanding of the variability in exposure measurements [23]. In the case of mycotoxins, and because they are a contaminant and not a chemical intentionally added or used in a process, there is a need to collect information about all the raw materials (e.g., flour, feed) in presence and their origin to understand if there is a need of measuring mycotoxins and, with this, pinpoint mycotoxins source in that workplace environment. This information is also very relevant to identify the risk management measures to put in place to avoid exposure.

An exposure assessment strategy should also characterize exposure variability (e.g., different levels along the work shift) and can be used for multiple purposes (e.g., baseline monitoring, evaluating new or in place risk management measures and to obtained epidemiological data). A common approach consists of measuring exposures due to individual tasks and using these data to estimate full-shift averages rather than measure full-shift averages directly. This task-based exposure assessment allows the evaluation of the contribution of specific tasks to overall exposures and supports the information where the risk management measures can have major impact in the overall exposure [23,24]. This approach might be relevant in the case of mycotoxins exposure since exposure through inhalation occurs essentially in tasks linked with high exposure to organic dust [20]. Probably those tasks should be the ones targeted for the implementation of risk management measures aiming to reduce exposure to organic dust.

Another approach, described also in EN 689:2020, is the constitution of similar exposure groups (SEGs) that can simplify significantly the exposure assessment if several workers have the same exposure profile. The SEG is composed by a group of workers having the same general exposure profile for the chemical agent(s) being studied because of the similarity and frequency of the tasks performed, the materials and processes with which they work, and the similarity of the way they perform the tasks. If several workers have the same exposure profile the measurements performed on some workers of the SEG will allow to conclude about exposure of all workers of the SEG. This approach can be used in both type of monitoring: environmental (air, raw materials) or biomonitoring.

Mycotoxins are a group of compounds with different toxicokinetic’ characteristics, namely the half-life in human body and excretion time. It has been referred that sampling could have an influence in the exposure assessment since significant differences were determined between mycotoxins’ urinary biomarkers levels when considering a 24 h urine sample or a first-morning urine sample [25]. These differences could have a higher impact when considering contaminants with short half-lives [26,27]. Recently, and regarding deoxynivalenol (DON), a biokinetic model was proposed with reference to a preferred collection of 24 h urine sample (or at least, a 12 h urine sample) for exposure assessment [28]. Thus, the sampling strategy (moment of sampling) should consider the toxicokinetic of each mycotoxin to allow the correct interpretation of the data and to better identify exposure sources (workplace or food consumption).

**Figure 1:**
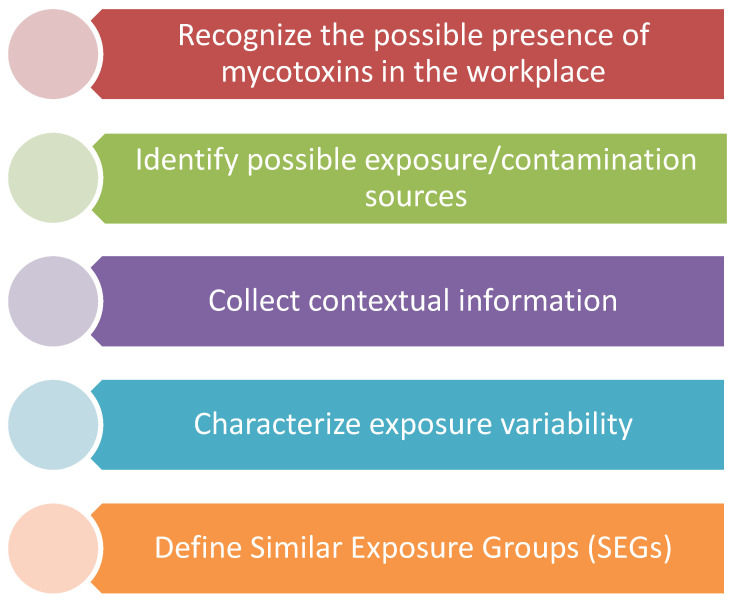
Key aspects to consider on the occupational exposure assessment to mycotoxins.

Figure 1 summarizes the key aspects that should be considered when occupational exposure assessment to mycotoxins is planned or performed.

## 3. Overview of Studies Developed in Portugal (2012–2020)

Several studies were already performed aiming to assess the occupational exposure to mycotoxins in different activity sectors from companies located in Portugal. Different factors trigger the development of these studies, either by the high presence of potential toxigenic fungal or by the presence of environmental factors (e.g., high exposure to organic dust) that can boost the mycotoxins exposure among other risk factors [20]. Additionally, the fact that mycotoxins are a relevant food contaminant, due to their common presence in the raw materials, raises the concern of possible exposure of workers from industries processing food commodities. Indeed, exposure in these types of industries was already reported [12,18,29].

In 2012, several field campaigns were performed in swine, focusing on fungal contamination on the barns, showing the higher prevalence of *Aspergillus* section *Nidulantes* [30]. However, other *Aspergillus* sections were also found, such as *Circumdati*, *Fumigati* and *Nigri*, all of them known by their toxigenic potential [31]. Twenty-one workers (75%) from those swine farms disclosed detectable exposure levels to aflatoxin B1 (AFB1) analyzed in blood samples, with values ranging from <1 ng/mL to 8.94 ng/mL and with a mean value of 1.91 ± 1.68 ng/mL. In the control group, the AFB1 values were all below 1 ng/mL (limit of detection, LOD). In this study, data demonstrated that exposure to AFB1 occurs in swine barns and that the workplace environment was the exposure source since the individuals from the considered control group (30 administrative workers from companies located in the same region) did not present detectable levels [10]. In a more recent study also developed in swine barns [13], the approach followed was different, considering several mycotoxins, analyzed in urine spot samples in addition to the evaluation of several environmental samples. Besides the biomonitoring campaign (workers and control group urine samples collection and analyses), several environmental samples (air, litter and feed) were also considered and analyzed for several mycotoxins. This approach allowed to reinforce that workers are exposed to several mycotoxins simultaneously and this was possible through the biomonitoring data and the high contamination (high concentrations and several mycotoxins in the same samples) found in feed and litter samples. The results demonstrated that the occupational environment was adding and contributing to the workers’ total exposure to mycotoxins, particularly in the case of DON and that feed and litter were relevant contamination sources of the workplace environment [13].

In addition, in 2012, a parallel study was developed in poultry production involving 31 poultry workers from six poultry farms and a control group (*n* = 30) from general population. Once again, the results obtained suggested that occupational exposure to AFB1 by inhalation occurs due to the high exposure to organic dust and represents an additional risk in this occupational setting that need to be recognized, assessed, and prevented [9]. The bases for developing this study was the high fungal contamination found and the fact that all the conditions needed for fungi proliferation were guaranteed in this workplace environment. In 2015, a study in a poultry slaughterhouse was developed aiming also to assess occupational exposure to AFB1. Despite uncertainties regarding the exposure route that was contributing more to exposure (inhalation or dermal) it was possible to conclude that exposure to AFB1 was occurring in the slaughterhouse. The findings obtained seemed to demonstrate that reducing AFB1 contamination in poultry production can result in preventing exposure in slaughterhouse environment [11].

Regarding the waste management industry, several efforts were developed since 2014 to assess the occupational exposure to mycotoxins. In this setting, the waste sorting for further waste processing was considered the most relevant due to the higher number of workers on the different workstations and the continuous handle of waste. In this case, mycotoxins were considered because, besides the fungal burden present, also the high nutrients availability due to the permanent presence of waste in the facilities was verified. Indeed, *Aspergillus* genera was the most prevalent genus on the waste sorting facilities assessed and *Aspergillus* section *Flavi* the third most prevalent [32]. Thus, biomonitoring campaigns in workers were developed, focused only in AFB1 exposure in a first attempt, and it was demonstrated that occupational exposure was occurring [33]. This was the beginning of a wider sampling campaign in the same industry, and through the biomonitoring data it was possible to confirm that exposure occurs for all the workers sampled, even for workers that are inside the equipment that move the waste mechanically (e.g., forklifts). It was also possible to conclude that the workplace environment was the main determinant of exposure since no detected exposure was found in the control group selected from the general population living nearby and without a professional activity related with waste management. Thus, in 2017 and further updated in 2020, we developed an additional study devoted to study the contamination present on the filters belonging to the filtration system from forklifts [34,35]. These filters were assessed and despite of the high fungal contamination found on filters (with *Aspergillus* section *Flavi* presenting lower prevalence than environmental sampling), none of the targeted mycotoxins (33 different mycotoxins were analyzed) were found in the aqueous filter extracts. The reason for this can be due to the constant air circulation in the filter that moves the particles of smaller diameter (carrying mycotoxins) into the vehicle cabinet not allowing the mycotoxins to be retained in the assessed filters [34,36]. Indeed, it is important to refer that the analyzed filters have pores dimensions typically of 3.0 μm or higher [37] and we need smaller pore sizes, namely 0.2 μm to retain mycotoxins [38,39,40]. Recently, in the same filters, a different study intending to assess the microbial composition (comprising fungi and bacteria) of complex aerosol mixtures was developed, applying high-throughput sequencing [35]. In the referred study, the detection by high-throughput sequencing of *Aspergillus* section *Flavi* as one of the most abundant fungal species corroborated the previous findings of occupational exposure to AFB1 in the same waste sorting unit [33]. Additionally, and to understand if besides inhalation other exposure routes should be consider, personal protection equipment (PPE) such as respiratory protection devices and gloves used by workers on the same waste sorting industry were collected and analyzed for fungi and mycotoxins contamination [35,41]. In both devices, the fungal burden obtained seems to mimic the contamination obtained by active air sampling [41,42]. However, only in gloves was it possible to detect mycotoxins, being the most reported the mycophenolic acid since 89.6% of the gloves samples analyzed present contamination by this mycotoxin. Therefore, both studies (biomonitoring and PPE) were important to recognize occupational exposure to mycotoxins and to identify the most critical workstations at the waste sorting industry and the respective sources of human exposure [33,42]. Additionally, the findings obtained with the PPE also raise the concern for possible mycotoxins exposure through ingestion in the workplace due to inadvertent hand-mouth contact.

Health care institutions are an occupational environment where mycotoxins contamination it’s not expected due to requirements concerning surfaces hygiene preventing fungi presence. However, in a study developed in 2019 in ten primary health care centers located in Portugal, toxigenic fungal species were found during the campaign and the lack of standardized hygiene routines claimed attention for the need to assess mycotoxins in this health care environment [43]. Furthermore, it was already reported the presence of mycotoxins in bioaerossols from one cancer treatment center [44]. In our study, besides air samples, mycotoxins were also detected in settled dust and heating, ventilation and air conditioning systems (HVAC) filters. Indeed, nine in forty one air samples (21%) were contaminated with 1 to 5 different mycotoxins in the same sample and the ones detected were fumonisins B1 (2 samples), B2 (6 samples,) and B3 (1 sample), roquefortine C (1 sample) and ochratoxin A (9 samples) and ochratoxin B (1 sample), being ochratoxin A (<0.6–2.25 ng/mL) the most prevalent and fumonisin B2 (<2.8–8.8 ng/mL) the mycotoxin with the highest measured values [43]. Concerning HVAC filters, four in twelve (33%) samples were contaminated with 1 and 2 mycotoxins in the same filter and the ones detected were fumonisin B2 (3 samples, 0.6–21.4 ng/g), ochratoxin A (1 sample, 6.7 ng/g), mycophenolic acid (1 sample, 40.3 ng/g) and sterigmatocystin (1 sample, <2.9 ng/g). In addition, in HVAC filters, fumonisin B2 was the most prevalent mycotoxin, exhibiting highest measured values. Three out of ten settled dust (30%) samples were contaminated by mycotoxins: one with three mycotoxins (roquefortine C griseofulvin and mycophenolic acid), and two with one mycotoxin each (mycophenolic acid and sterigmatocystin) [45]. Overall, the sampling approach followed allowed to recognize the possible exposure to mycotoxins in health care facilities for patients and workers [43]. Further studies aiming to charaterize workers’ exposure is still needed with the inclusion of biological samples collection to prove if the presence of mycotoxins in environmental samples represent or not a real source of exposure to individuals (workers or patients).

Due to raw materials used in bakeries facilities (e.g., flour) that have already showed contamination in several reports [46,47,48], mycotoxins presence in the work environment was also expected. Indeed, in a study developed in 14 Portuguese bakeries located in the Lisbon district it was possible to detect mycotoxins on all the 11 settled dust samples analyzed and DON was clearly the mycotoxin measured in higher amounts. However, in the same study, mycotoxins were not detected in air samples collected in the same facilities (active sampling methods) [49]. Thus, the use of passive sampling methods such as settled dust appears to be the best option for mycotoxins assessment in occupational environments [18,50,51]. Interestingly, settled dust did not show any fungal growth supporting the evidence that the indoor presence of fungal species does not imply the exposure to mycotoxins and vice versa. In line with the results obtained in the settled dust samples of this study, a new study developed in a fresh bread dough company using biomonitoring data from workers also showed that workplace exposure added significantly to the exposure resulting from ingestion of mycotoxin-contaminated food, particularly in the case of DON due to the flour contamination and the frequent manual handling [12]. Accordingly, in the settled dust sample also collected in this company, DON was the mycotoxin measured in high amounts (58.2 ng/g), corroborating the role of the workplace environment as exposure source [12].

More recently, in a study developed in one dairy farm, all the feed samples analyzed presented contamination by at least two mycotoxins and up to a maximum of 13 mycotoxins in the same sample. Zearalenone (ZEA) was detected in all the samples (*n* = 10, 0.60–266 ng/g) followed by DON (*n* = 8, <1–197 ng/g), and Ochratoxin A (OTA) (*n* = 5, <0.13–4.53 ng/g) [14]. Therefore, and in line with the results previously found in swine, feed can be the principal contamination source for mycotoxins in all occupational environments where animals need to be fed [13]. In this, and in previous studies developed in animal production and food processing facilities, besides an occupational health concern, there is also a food safety concern present—justifying a holistic approach to prevent all these concerns. An adequate One Health approach should address this perspective through effective preventive actions such as avoiding the use of contaminated raw materials (e.g., feed, flours) [14].

Besides the key issues to consider to perform an occupational exposure assessment to mycotoxins (Figure 1), the results obtained in the previous studies performed (Table S1) need to be reflect and ponder how they might support decision for defining a sampling strategy.

Whereas passive-collection methods (e.g., settled dust) allow the collection of contamination over a longer period (days, weeks or several months), air samples can only reflect the load of a shorter period (mostly minutes) corresponding to the sampling duration [13,45,52]. Passive and active methods should, therefore, be used in parallel to ensure a more precise assessment of occupational exposure to bioburden [45,52]. Increasing the number of different sampling methods will increase and enrich the data, enabling industrial hygienists to identify contamination sources and perform a more accurate risk characterization and management [13,45,52].

The collection and analysis of settled dust samples from indoor environments has become one of several environmental sampling methods used during bioburden evaluations by several researchers [53,54]. Indeed, the option for collecting settled dust seems a good approach also for the assessment of mycotoxins contamination of the workplace environment [13,55,56].

Human biomonitoring (HBM) is an important tool for assessing directly the exposure to mycotoxins and it is being more commonly used in the recent years to assess exposure of general population [25,57,58,59] and workers [60,61,62]. However, it is important to note that data on background dietary exposure to mycotoxins is needed to determine the additional burden of respiratory and dermal exposure in the workplace [63] since we are dealing with an important food contaminant. If this background data are unavailable, a control group of individuals from the general population should be included to exclude the possibility of workers exposure by food consumption [61,63]. Indeed, most of the HBM studies developed in workplaces aiming to assess exposure to mycotoxins had included a control group, usually engaging workers from administrative companies in the same locality, which enables to take into account the exposure by food intake and a better understanding of the role of working environments in the total burden of mycotoxin exposure [20]. This approach allows us to understand which workplace adds to the exposure occurring by way of food intake. In order to identify the most important exposure sources as a basis for selection of the most relevant risk management measures and controlling emissions at source, HBM data on internal exposure should be combined with contextual data. The information on individual behaviors in the workplace (e.g., the use of PPE), diet, workplace environment, lifestyle, monitoring data about environmental and/or food contamination, and sociodemographic data is a contribute to improve the accuracy of subsequent analysis and conclusions since it allow to disclose the possible influence of sociodemographic determinants of exposure. This was evidenced in a study developed in the poultry slaughterhouse where dermal absorption was identified as an exposure route due to details of a specific workplace obtained during the company walkthrough visit done by the occupational hygienist [11]. This contextual information can be even more refined if including environmental samples collected from the workplace. These samples should be selected to understand from where contamination is coming in a specific workplace environment and main sources of workplace contamination. This was also the approach followed in the more recent studies where it was possible to recognize that the use of contaminated raw materials (e.g., feed and flour) brings contamination for the workplace environment. Moreover, and more recently, the use of PPE as sampling matrixes in the waste management settings also allow to recognize other exposure routes that were not identified in a first approach (e.g., ingestion through hand-mouth contact). Additionally, assessing exposure to several mycotoxins simultaneously in biological and environmental samples is more adequate than analyzing only one mycotoxin since exposure occurs as a mixture of mycotoxins, for both workers and general population. This is understandable since, besides the presence of multiple mycotoxins in the workplace environment, this is also a common feature of raw materials and food commodities [20,61].

## 4. Recommendations for Future Studies and Directions

Previous studies reported that fungal contamination indoors depends on activities performed by indoor occupants [64,65], ventilation, building design, environmental characteristics [66], water infiltrations and damage [67], and by the geographical location and climate conditions [68]. As such, due to this wide array of factors influencing microbial contamination indoors, passive methods are probably more reliable than active methods (air sampling) to characterize fungal contamination, and covering also mycotoxins, since they can collect contamination from a longer period (one workshift, days, weeks or even months), thus, covering all the possible variations [45]. Furthermore, the collection and analysis of settled dust samples is one of several environmental sampling methods used for mycotoxins contamination/exposure assessments [55,56]. Indeed, that was the case with some of the studies performed and presented on Table S1.

Additionally, climate change (CC) scenarios are expected to have significant effects on the security and safety of food commodities and, consequently, also in exposure occurring in the workplaces. Indeed, one of the most relevant factors that can be influenced by CC is the infection of crops by mycotoxigenic molds and, the subsequent contamination with mycotoxins [69,70,71]. Therefore, considering that workers in many workplaces might need to handle contaminated materials such as raw materials for food production, the increase of contamination can result in higher exposure levels for workers. Therefore, risk management measures targeted for reducing mycotoxins contamination in food (e.g., raw materials selection based on reduced contamination by mycotoxins) will have a positive effect in controlling workers’ exposure. However, and since CC is already in place, more holistic interventions might be needed concerning mitigation and adaptation to CC at international level.

There is also a new challenge: the concept of a circular economy in the EU and what can represent regarding mycotoxins occupational exposure. The European Commission has expressed the importance of a circular economy as follows: ‘The transition to a more circular economy—in which the value of products, materials and resources can be retained for as long as possible in the economy and waste production is kept to a minimum makes a vital contribution to the efforts of the EU to foster a sustainable, low-carbon, resource-efficient and competitive economy,’ [72]. In this, the European Commission published its proposal for a circular economy action plan, which also included a plan for cutting food and agricultural waste. Although the transition to a circular economy provides opportunities, including for the agro-food sector, also brings some concerns due to the intended use of residue streams from the agricultural sector for biogas production and, through refinery, into other high-value products, such as compost, animal feed and biodiesel. Indeed, a circular economy has the potential to address several targets of the sustainable development goals in Agenda 2030, relating to human health and the environment, sustainable management of natural resources as well as creation of jobs and economic growth. However, this will certainly imply several workers involved in the handle of residues as raw material and, in the process end, as final product representing additional exposure scenarios where occupational exposure to mycotoxins can occur.

New studies should be developed aiming to answer to these emerging challenges and to prevent knowledge gaps. This will certainly help to regulate better and to act to prevent mycotoxins exposure of workers and consumers.

## Supplementary Materials

The following are available online at https://www.mdpi.com/2072-6651/12/8/513/s1, Table S1: Overview of Studies Developed in Portugal (2012–2020).

## References

[1] Burg W.A., Shotwell O.L., Saltzman B.E. (1981). Measurements of airborne aflatoxins during the handling of contaminated corn. Am. Ind. Hyg. Assoc. J..

[2] Fischer G., Müller T., Ostrowski R., Dott W. (1999). Mycotoxins of Aspergilius fumigatus in pure culture and in native bioaerosols from compost facilities. Chemosphere.

[3] Skaug M.A., Eduard W., Stormer F.C. (2000). Ochratoxin A in airborne dust and fungal conidia. Mycopathologica.

[4] Nordby K.C., Halstensen A.S., Elen O., Clasen P.-E., Langseth W., Kristensen P., Eduard W. (2004). Trichothecene mycotoxins and their determinants in settled dust related to grain production. Ann. Agric. Environm. Med..

[5] Wanga Z., Chai T., Lu G., Quan C., Duan H., Yao M., Zucker B.-A., Schlenker G. (2008). Simultaneous detection of airborne Aflatoxin, Ochratoxin and Zearalenone in a poultry house by immunoaffinity clean-up and high-performance liquid chromatography. Environ. Res..

[6] Lanier C., Richard E., Heutte N., Picquet R., Bouchart V., Garon D. (2010). Airborne molds and mycotoxins associated with handling of corn silage and oilseed cakes in agricultural environment. Atmos. Environ..

[7] Mastanjević K., Lukinac J., Jukić M., Šarkanj B., Krstanović V., Mastanjević K. (2019). Multi-(myco)toxins in Malting and Brewing By-Products. Toxins.

[8] Mastanjević K., Šarkanj B., Krska R., Sulyok M., Warth B., Mastanjević K., Šantek B., Krstanović V. (2018). From malt to wheat beer: A comprehensive multi-toxin screening, transfer assessment and its influence on basic fermentation parameters. Food Chem..

[9] Viegas S., Veiga L., Malta-Vacas J., Sabino R., Figueiredo P., Almeida A., Viegas C., Carolino E. (2012). Occupational exposure to aflatoxin (afb1) in poultry production. J. Toxicol. Environ. Health Part A.

[10] Viegas S., Veiga L., Figueiredo P., Almeida A., Carolino E., Sabino R., Veríssimo C., Viegas C. (2013). Occupational exposure to aflatoxin B1 in swine production and possible contamination sources. J. Toxicol. Environ. Health Part A.

[11] Viegas S., Veiga L., Almeida A., dos Santos M., Carolino E., Viegas C. (2015). Occupational Exposure to Aflatoxin B1 in a Portuguese Poultry Slaughterhouse. Ann. Occup. Hyg..

[12] Viegas S., Assunção R., Nunes C., Osteresch B., Twaruzek M., Kosicki R., Grajewski J., Martins C., Alvito P., Almeida A. (2018). Exposure Assessment to Mycotoxins in a Portuguese Fresh Bread Dough Company by Using a Multi-Biomarker Approach. Toxins.

[13] Viegas S., Assunção R., Martins C., Nunes C., Osteresch B., Twarużek M., Kosicki R., Grajewski J., Ribeiro E., Viegas C. (2019). Occupational exposure to mycotoxins in swine production: Environmental and biological monitoring approaches for exposure assessment. Toxins.

[14] Viegas S., Assunção R., Twaruźek M., Kosicki R., Grajewski J., Viegas C. (2019). Mycotoxins feed contamination in a dairy farm—Potential implications for milk contamination and workers’ exposure in a One Health approach. J. Sci. Food Agric..

[15] Selim M., Juchems A.M., Popendorf W. (1998). Assessing Airborne Aflatoxin B1 during On-Farm Grain Handling Activities. Am. Ind. Hyg. Assoc. J..

[16] Oluwafemi E., Odebiyi T., Kolapo A. (2012). Occupational aflatoxin exposure among feed mill workers in Nigeria. World Mycotoxin J..

[17] Mayer S., Vishwanath V., Sulyok M., Johanning E., Morrey P.R., Auger P. (2012). Airborne Workplace Exposure to Microbial Metabolites in Waste Sorting Plants.

[18] Straumfors A., Uhlig S., Eriksen G.S., Heldal K.K., Eduard W., Krska R., Sulyok M. (2014). Mycotoxins and other fungal metabolites in grain dust from Norwegian grain elevators and compound feed mills. World Mycotoxin J..

[19] Malik A., Ali S., Shahid M., Bhargava R. (2014). Occupational exposure to Aspergillus and aflatoxins among food-grain workers in India. Int. J. Occup. Environ. Health.

[20] Viegas S., Viegas C., Oppliger O. (2018). Occupational Exposure to Mycotoxins: Current Knowledge and Prospects. Ann. Work Expo. Health.

[21] Assunção R., Silva M.J., Alvito P. (2016). Challenges in risk assessment of multiple mycotoxins in food. World Mycotoxin J..

[22] Chen C.-C., Chuang Kuen C.-L., Wu Y., Chan C.-C. (2009). Sampling Strategies for Occupational Exposure Assessment under Generalized Linear Model. Ann. Occup. Hyg..

[23] Ramachandran G. (2008). Toward Better Exposure Assessment Strategies—The New NIOSH Initiative. Ann. Occup. Hyg..

[24] Viegas S., Almeida-Silva M., Faria T., Dos Santos M., Viegas C., Arezes P.M., Perestrelo G., Miguel S., Melo R.B. (2016). Occupational exposure assessment to particles with task-based approach. Occupational Safety and Hygiene IV.

[25] Martins C., Vidal A., De Boevre M., De Saeger S., Nunes C., Torres D., Goios A., Lopes C., Assuncao R., Alvito P. (2019). Exposure assessment of Portuguese population to multiple mycotoxins: The human biomonitoring approach. Int. J. Hyg. Environ. Health.

[26] Aylward L.L., Sean M.H., Smolders R., Koch H.M., Cocker J., Jones K., Warren N., Levy L., Bevan R. (2014). Sources of Variability in Biomarker Concentrations. J. Toxicol. Environ. Health Part B.

[27] Calafat A.M. (2016). Contemporary Issues in Exposure Assessment Using Biomonitoring. Curr. Epidemiol. Rep..

[28] Mengelers M., Zeilmaker M., Vidal A., De Boevre M., De Saeger S., Hoogenveen R. (2019). Biomonitoring of Deoxynivalenol and Deoxynivalenol-3-glucoside in Human Volunteers: Renal Excretion Profiles. Toxins.

[29] Viegas C., Pacífico C., Faria T., Cebola de Oliveira A., Aranha Caetano L., Carolino E., Quintal Gomes A., Viegas S. (2017). Fungal contamination in green coffee beans. J. Toxicol. Environ. Health A.

[30] Viegas C., Carolino E., Sabino R., Viegas S., Veríssimo C. (2013). Fungal Contamination in Swine: A Potential Occupational Health Threat. J. Toxicol. Environ. Health Part A..

[31] Varga J., Baranyi N., Chandrasekaran M., Vágvölgyi C., Kocsubé S. (2015). Mycotoxin producers in the Aspergillus genus: An update. Acta Biol. Szeged..

[32] Viegas C., Faria T., dos Santos M., Carolino E., Quintal Gomes A., Sabino R., Viegas S. (2015). Fungal burden in waste industry: An occupational risk to be solved. Environ. Monit. Assess.

[33] Viegas S., Veiga L., Figueiredo P., Almeida A., Carolino E., Viegas C. (2014). Assessment of Workers’ Exposure to Aflatoxin B1 in a Portuguese Waste Industry. Ann. Occup. Hyg..

[34] Viegas C., Faria T., Cebola de Oliveira A., Aranha Caetano L., Carolino E., Quintal-Gomes A., Twarużek M., Kosicki R., Soszczyńska E., Viegas S. (2017). A new approach to assess fungal contamination and mycotoxins occupational exposure in forklifts drivers from waste sorting. Mycotoxin Res..

[35] Viegas C., Caetano L.A., Cox J., Korkalainen M., Haines S.R., Dannemiller K.C., Viegas S., Reponen T. (2020). The effects of waste sorting in environmental microbiome, THP-1 cell viability and inflammatory responses. Environ. Res..

[36] Viegas C., Monteiro A., dos Santos M., Faria T., Aranha Caetano L., Carolino E., Quintal-Gomes A., Marchand G., Lacombe N., Viegas S. (2018). Filters from taxis air conditioning system: A tool to characterize driver’s occupational exposure to bioburden?. Environ. Res..

[37] Capacci E., Rondelli V. Tractor cab to protect the operator from hazardous sub-stances in spray application. Proceedings of the International Conference of Agricultural Engineering.

[38] Johanning E., Gareis M., Nielsen K., Dietrich R., Märtlbauer E. Airborne mycotoxin sampling and screening analysis. Proceedings of the Indoor Air 2002, the 9th International Conference on Indoor Air Quality and Climate.

[39] Kildesø J., Wurtz H., Nielsen K.F., Wilkins C.K., Gravesen S., Nielsen P.A., Thranev U., Schneider T., Seppanen O., Sateri J. (2000). The release of fungal spores from water damaged building materials. Proceedings of the Healthy Buildings 2000.

[40] Pasanen A.L., Nikulin M., Tuomainen M., Parikka P., Hintikka E.L. (1993). Laboratory experiments on membrane filter sampling of airborne mycotoxins produced by Stachybotrys atra Corda. Atmos. Environ..

[41] Viegas C., Dias M., Almeida B., Aranha Caetano L., Carolino E., Quintal Gomes A., Twaruzek M., Kosicki R., Grajewski J., Marchand G. (2020). Are workers from waste sorting industry really protected by wearing Filtering Respiratory Protective Devices? The gap between the myth and reality. Waste Manag..

[42] Viegas C., Twarużek M., Dias M., Almeida B., Carolino E., Kosicki R., Soszczyńska E., Grajewski J., Aranha Caetano L., Viegas S. (2020). Assessment of the microbial contamination of mechanical protection gloves used on waste sorting industry: A contribution for the risk characterization. Environ. Res..

[43] Viegas C., Almeida B., Monteiro A., Aranha Caetano L., Carolino E., Quintal-Gomes A., Twarużek M., Kosicki R., Marchand G., Viegas S. (2019). Bioburden in healthcare centers: Is the compliance with Portuguese legislation enough to prevent and control infection?. Build. Environ..

[44] Heutte N., Andre V., Dubos Arvis C., Bouchart V., Lemarie F., Legendre P., Votier E., Louis M.-Y., Madelaine S., Séguin V. (2017). Assessment of multi-contaminant exposure in a cancer treatment center: A 2-year monitoring of molds, mycotoxins, endotoxins, and glucans in bioaerosols. Environ. Monit. Assess.

[45] Viegas C., Almeida B., Monteiro A., Paciência I., Cavaleiro J.R., Carolino E., Quintal-Gomes A., Twarużek M., Kosicki R., Marchand G. (2019). Settled dust assessment in clinical environment: Useful for the evaluation of a wider bioburden spectrum. Int. J. Environ. Health Res..

[46] Bergamini E., Catellani D., Dall’asta C., Galaverna G., Dossena A., Marchelli R., Suman M. (2010). Fate of fusarium mycotoxins in the cereal product supply chain: The deoxynivalenol (DON) case within industrial bread-making technology. Food Addit. Contam. Part A.

[47] Belluco B., de Camargo A.C., da Gloria E.M., dos Santos Dias C.T., Button D.C., Calori-Domingues M.A. (2017). Deoxynivalenol in wheat milling fractions: A critical evaluation regarding ongoing and new legislation limits. J. Cereal Sci..

[48] Schaarschmidt S., Fauhl-Hassek C. (2018). The Fate of Mycotoxins during the Processing of Wheat for Human Consumption. Compr. Rev. Food Sci. Food Saf..

[49] Viegas C., Faria T., Aranha Caetano L., Carolino E., Quintal-Gomes A., Twaruzek M., Kosicki R., Viegas S. (2019). Characterization of Occupational Exposure To Fungal Burden in Portuguese Bakeries. Microorganisms.

[50] Mo X., Lai H., Yang Y., Xiao J., He K., Liu C., Chen J., Lin Y. (2014). How does airway exposure of aflatoxin B1 affect serum albumin adduct concentrations? Evidence based on epidemiological study and animal experimentation. J. Toxicol. Sci..

[51] Lai H., Mo X., Yang Y., He K., Xiao J., Liu C., Chen J., Lin Y. (2014). Association between aflatoxin B1 occupational airway exposure and risk of hepatocellular carcinoma: A case-control study. Tumor Biol..

[52] Viegas C., Faria T., Monteiro A., Aranha Caetano L., Carolino E., Quintal Gomes A., Viegas S. (2018). A Novel Multi-Approach Protocol for the Characterization of Occupational Exposure to Organic Dust—Swine Production Case Study. Toxics.

[53] Leppänen H.K., Täubel M., Jayaprakash B., Vepsäläinen A., Pasanen P., Hyvärinen A. (2018). Quantitative assessment of microbes from samples of indoor air and dust. J. Expo. Sci. Environ. Epidemiol..

[54] Park J.-H., Sulyok M., Lemons A.R., Green B.J., Cox-Ganser J.M. (2018). Characterization of fungi in office dust: Comparing results of microbial secondary metabolites, fungal internal transcribed spacer region sequencing, viable culture and other microbial indices. Indoor Air.

[55] Halstensen A.S., Nordby K.-C., Eduard W., Klemsdal S.S. (2006). Real-time PCR detection of toxigenic Fusarium in airborne and settled grain dust and associations with trichothecene mycotoxins. J. Environ. Monit..

[56] Tangni E.K., Pussemier L. (2007). Ergosterol and mycotoxins in grain dusts from fourteen Belgian cereal storages: A preliminary screening survey. J. Sci. Food Agric..

[57] Heyndrickx E., Sioen I., Huybrechts B., Callebaut A., De Henauw S., De Saeger S. (2015). Human biomonitoring of multiple mycotoxins in the Belgian population: Results of the BIOMYCO study. Environ. Int..

[58] Sarkanj B., Ezekiel C.N., Turner P.C., Abia W.A., Rychlik M., Krska R., Sulyuk M., Warth B. (2018). Ultra-sensitive, stable isotope assisted quantification of multiple urinary mycotoxin exposure biomarkers. Anal. Chim. Acta.

[59] Zhang S., Zhou S., Gong Y.Y., Zhao Y., Wu Y. (2020). Human dietary and internal exposure to zearalenone based on a 24-hour duplicate diet and following morning urine study. Environ. Int..

[60] Föllmann W., Ali N., Blaszkewicz M., Degen G.H. (2016). Biomonitoring of Mycotoxins in Urine: Pilot Study in Mill Workers. J. Toxicol. Environ. Health Part A.

[61] Viegas S., Martins C. (2020). The Usefulness of Human Biomonitoring in the Case of Mycotoxins Exposure Assessment.

[62] Debegnach F., Brera C., Mazzilli G., Sonego E., Buiarelli F., Ferri F., Rossi P.G., Collini G., De Santis B. (2020). Optimization and validation of a LC-HRMS method for aflatoxins determination in urine samples. Mycotoxin Res..

[63] Degen G.H. (2008). The challenge to assess workplace related risks from mycotoxin exposure. Mycotoxin Res..

[64] Täubel M., Rintala H., Pitkäranta M., Paulin L., Laitinen S., Pekkanen J., Hyvärinen A., Nevalainen A. (2009). The occupant as a source of house dust bacteria. J. Allergy Clin. Immunol..

[65] Lax S., Smith D.P., Hampton-Marcell J., Owens S.M., Handley K.M., Scott N.M., Gibbons S.M., Larsen P., Shogan B.D., Weiss S. (2014). Longitudinal analysis of microbial interaction between humans and the indoor environment. Science.

[66] Meadow J.F., Altrichter A.E., Kembel S.W., Kline J., Mhuireach G., Moriyama M., Northcutt D., O’Connor T.K., Womack A.M., Brown G.Z. (2014). Indoor airborne bacterial communities are influenced by ventilation, occupancy, and outdoor air source. Indoor Air.

[67] Emerson J.B., Keady P.B., Brewer T.E., Clements N., Morgan E.E., Awerbuch J., Miller S.L., Fierer N. (2015). Impacts of flood damage on airborne bacteria and fungi in homes after the 2013 Colorado front range flood. Environ. Sci. Technol..

[68] Barberán A., Dunn R.R., Reich B.J., Pacifici K., Laber E.B., Menninger H.L., Morton J.M., Henley J.B., Leff J.W., Miller S.L. (2015). The ecology of microscopic life in household dust. Proc. R. Soc. B Biol. Sci..

[69] Assunção R., Martins C., Viegas S., Viegas C., Jakobsen L.S., Pires S., Alvito P. (2018). Climate change and the health impact of aflatoxins exposure in Portugal–an overview. Food Addit. Contam. Part A.

[70] Assunção R., Vettorazzi A., González-Peñas E., Martins C. (2020). Climate Change and Aflatoxins Contamination in the Iberian Peninsula. Reference Module in Life Sciences.

[71] Medina A., Akbar A., Baazeem A., Rodriguez A., Magan N. (2017). Climate change, food security and mycotoxins: Do we know enough?. Fungal Biol. Rev..

[72] European Commission (2015). Circular Economy Package: Q&A.

