# Supplementary material for: Occupational Exposure to Mycotoxins—Different Sampling Strategies Telling a Common Story Regarding Occupational Studies Performed in Portugal (2012–2020)
Source: Toxins (Basel). 2020 Aug 11;12(8):513. doi: 10.3390/toxins12080513 (PMC7472215; doi:10.3390/toxins12080513)
Supplement: Supplementary file 1 [file toxins-12-00513-s001.pdf]

Supplementary Materials: Occupational Exposure to Mycotoxins—Different Sampling Strategies Telling a Common Story Regarding Occupational Studies Performed in Portugal (2012–2020)

Susana Viegas, Carla Viegas, Carla Martins and Ricardo Assunção

Table S1. Overview of Studies Developed in Portugal (2012–2020)

Occupational Environment	Type of Samples (Matrix)	Mycotoxins Analyzed	Results	Main Conclusions Concerning Exposure	References
Swine	HBM (blood samples) from workers (*n* = 28) and a control group (*n* = 30) subjects without any type of agricultural activity.	Aflatoxin B1 (AFB1)	Twenty-one workers (75%) showed detectable levels of AFB1 with values ranging from <1 ng /mL to 8.94 ng/mL and with a mean value of 1.91 ± 1.68 ng/mL. In the control group, the AFB1 values were all below 1 ng/mL.	Data indicate that exposure to AFB 1 occurs in swine barns, and this site serves as a contamination source in an occupational setting.	[1]
	HBM ((urine) samples from workers (*n* = 25)<br><br>38 environmental samples (air samples, *n* = 23; litter samples, *n* = 5; feed samples, *n* = 10)	aflatoxin M1 (AFM1), aflatoxin B1 (AFB1), aflatoxin B2 (AFB2), aflatoxin G1 (AFG1), aflatoxin G2 (AFG2), patulin (PAT), nivalenol (NIV), deoxynivalenol (DON), deoxynivalenol-3-glucoside (DON-3-G), 15-acetyldeoxynivalenol (15-AcDON), 3-acetyldeoxynivalenol (3-AcDON), deepoxy-deoxynivalenol (DOM-1), deoxynivalenol-glucuronide (DON-GlcA), fusarenon-X (FUS-X), α-zearalanol (α-ZAL), β-zearalanol (β-ZAL), α-zearalenol (α-ZEL), β-zearalenol (β-ZEL), zearalenone (ZAN), zearalenone (ZEN), toxin T-2 (T-2), toxin HT-2 (HT-2), toxin HT-2-4-glucuronide (HT-2-4-GlcA), T-2 tetraol, T-2 triol, neosolaniol (NEO), monoacetoxyscirpenol (MAS), diacetoxyscirpenol (DAS), fumonisin B1 (FB1), fumonisin B2 (FB2), fumonisin B3 (FB3), roquefortine C (ROQ-C), griseofulvin (GRIS), ochratoxin A (OTA), ochratoxin B (OTB), ochratoxin alpha (OTα), mycophenolic acid (MPA), mevinolin (MEV), sterigmatocystin (STER), citrinin (CIT), dihydrocitrinone (DH-CIT), Enniatin B (EnB),	The mycotoxins biomarkers detected in the urine samples of the workers group were the deoxynivalenol-glucuronic acid conjugate (60%), aflatoxin M1 (16%), enniatin B (4%), citrinin (8%), dihydrocitrinone (12%) and ochratoxin A (80%). Results of the control group followed the same pattern, but in general with a lower number of quantifiable results (<LOQ).<br><br>Only 3 air samples from two different farms showed contamination by sterigmatocystin (STER) (<LOQ–1.42 ng/g). All the other air samples were found to be negative for In litter samples prevalent mycotoxins were DON (<LOQ–76.4 ng/g) and STER (1.14–2.69 ng/g) which were detected in all litter samples and in considerably higher amounts than the other	Occupational environment is adding and contributing to the workers' total exposure to mycotoxins, particularly in the case of DON.<br>Workers and general population are exposed to several mycotoxins simultaneously.<br>Occupational exposure is probably described as being intermittent and with very high concentrations for short durations.	[2]

			analyzed mycotoxins. Zearalenone was a mycotoxin that was also detected in 4 out of 5 farms, but in lower amounts (<LOQ–0.78 ng/g). In feed samples, it was observed co-occurrence of mycotoxins in the same sample (9–17 mycotoxins were detected in the same sample). The higher values were obtained for DON (values between 137–388 ng/g) and fumonisins, particularly FB1 (values between 6–366 ng/g). Others mycotoxins, such as ZEN, 3-AcDON, 15-AcDON, and DON-3-G, fumonisins (FB1, FB2 and FB3), and type A trichothecenes such as T-2 and HT-2, were also detected in almost all the feed samples.		
Poultry	HBM (Blood samples) poultry workers (*n* = 31) and a control group (*n* = 30) workers who undertook administrative tasks.	Aflatonin B1 (AFB1)	Eighteen poultry workers (59%) had detectable levels of AFB1 with values ranging from <1 ng /mL to4.23 ng/mL and with a mean value of 2 ± 0.98 ng/mL. AFB1 was not detected in the serum sampled from any of the controls.	Data indicate that AFB1 inhalation represents an additional risk that needs to be recognized, assessed, and prevented.	[3]
Poultry slaugtherhouses	HBM (blood) workers (*n* = 30) and control group (*n* = 30)	Aflatoxin B1 (AFB1)	Fourteen workers (47.0%) showed detectable levels of AFB1 with values from 1.06 to 4.03 ng mL^{-1}, with a mean value of 1.73 ng mL^{-1}. No AFB1 was detected in serum of individuals used as controls.	Occupational exposure to AFB1 is occurring in the slaughterhouse studied.	[4]
Waste sorting	Eleven fork lifters filters	aflatoxin G2, aflatoxin G1, aflatoxin B2, aflatoxin B1,	No mycotoxins were	Further research is required to check if the	[5]

	agroclavin, eoxynivalenol, deoxynivalenol-3-glucoside, nivalenol, fusarenon X, deepoxy-deoxynivalenol, 3-acetyldeoxynivalenol, neosolaniol, noacetoxyscirpenol, diacetoxyscirpenol, HT-2 toxin, T-2 toxin, beauvericin, enniatin B, enniatin B1, enniatin A1, enniatin A, hydrolyzed fumonisin B1, fumonisin B1, fumonisin B2, ergovalin, dihydroergosin, ergotamin, ergocornin, moniliformin, patulin, ochratoxin α, ochratoxin B, ochratoxin A, verrucarin A, verrucarol, zearalenone-4-glucoside, α-zearalenol, βzearalenol, zearalenone-4-sulfate, and zearalenone	detected	environmental conditions as present in the filters could allow the production of mycotoxins and their dissemination in the cabinet during the normal use of the vehicles	
Filtering respiratory protection devices (FRPD) (*n* = 120) (both in interior layers and in exhalation valves)	15-Acetyldeoxynivalenol, 3-Acetyldeoxynivalenol, Aflatoxin B1, Aflatoxin B2 Aflatoxin G1, Aflatoxin G2 Aflatoxin M1, α-Zearalanol α-Zearalenol, β-Zearalanol, β-Zearalenol, Deepoxydeoxynivalenol, Deoxynivalenol, Diacetoxyscirpenol, DON Glucosid, Fumonisin B1, Fumonisin B2, Fumonisin B3, Fusarenon-X, Gliotoxin Griseofulvin, HT-2 Toxin Mevinolin, Moniliformin, Monoacetoxyscirpenol, Mycophenolic acid, Neosolaniol, Nivalenol, Ochratoxin A, Ochratoxin B, Patulin, Roquefortine C, Sterigmatocystin, T-2 Tetraol, T-2 Toxin, T-2 Triol Zearalanone, Zearalenon	No mycotoxins were detected.	Mycotoxins were not detected on none of the matrixes from none FRPD. This can be due to several reasons such as: (a) the fungi found were not able to produce mycotoxins; (b) the analytical method used was not capable to detect vestigial concentrations of mycotoxins, (c) the exterior layer of the FRPD is effective in protecting from particles that are the main carriers of mycotoxins for the workers respiratory system.	[6]
Mechanic protection gloves (MPG) (*n* = 67)	15-Acetyldeoxynivalenol, 3-Acetyldeoxynivalenol, Aflatoxin B1, Aflatoxin B2 Aflatoxin G1, Aflatoxin G2 Aflatoxin M1, α-Zearalanol α-Zearalenol, β-Zearalanol, β-Zearalenol, Deepoxydeoxynivalenol, Deoxynivalenol, Diacetoxyscirpenol, DON Glucosid, Fumonisin B1, Fumonisin B2, Fumonisin B3, Fusarenon-X, Gliotoxin Griseofulvin, HT-2 Toxin Mevinolin, Moniliformin, Monoacetoxyscirpenol, Mycophenolic acid, Neosolaniol, Nivalenol, Ochratoxin A, Ochratoxin B, Patulin, Roquefortine C, Sterigmatocystin, T-2 Tetraol, T-2 Toxin, T-2 Triol Zearalanone, Zearalenon	Mycotoxins were detected in 89.6% (60 out of 67) MPG samples. Seven mycotoxins were detected: neosolaniol in two samples (<LOQ), monoacetoxyscirpenol in one sample (19.2 μg/Kg), diacetoxyscirpenol also in one sample (25.0 μg/Kg), roquefortine C in twenty nine samples (<LOQ – 69.3 μg/Kg), griseofulvin also detected in twenty nine samples (<LOQ – 34.9 μg/Kg), mycophenolic acid in sixty samples (<LOQ – 105.6 μg/Kg) and	Mycotoxins were detected in 89.6% of the MPG. MPG can be used as screening method to identify the most critical workstations where Occupational Health multiple interventions should be prioritized.	[7]

			sterigmatocystin in two samples (<LOQ).). The most reported mycotoxin was mycophenolic acid (89.6%) followed by roquefortine C (43.3%) and griseofulvin (43.3%). Most MPG samples presented only one type of mycotoxin (mycophenolic acid), with the maximum number of mycotoxins per MPG sample being four mycotoxins.		
	HBM (blood) Workers (*n* = 41) and controls (*n* = 30)	Aflatoxin B1 (AFB1)	All the workers showed detectable levels of AFB1 with values ranging from 2.5 ng/mL to 25.9 ng/mL with a median value of 9.9 ± 5.4 ng/mL. All of the controls showed values below the method's detection limit.	The data obtained suggests that exposure to AFB1 occurs in a waste management setting and claims attention for the need of appliance of preventive and protective safety measures.	[8]
Primary Health Care Centers	Settled dust (*n* = 10)	Patulin, nivalenol, deoxynivalenol-3-glucoside, deoxynivalenol, fusarenon-X, α-zearalanol, β-zearalanol, β-zearalenol, α-zearalenol, zearalanone, zearalenone, T2 tetraol, deepoxydeoxynivalenol, neosolaniol, 15-acetyldeoxynivalenol, 3-acetyldeoxynivalenol, monoacetoxyscirpenol, diacetoxyscirpenol, aflatoxin M1, aflatoxin B1, aflatoxin B2, aflatoxin G1, aflatoxin G2, fumonisin B1, fumonisin B2, fumonisin B3, T2 triol, roquefortine C, griseofulvin, T2 toxin, HT2 toxin, ochratoxin A, ochratoxin B, mycophenolic acid, mevinolin and sterigmatocystin.	Three out of ten settled dust samples were contaminated by mycotoxins: one, the PHCC 9, with three mycotoxins (roquefortine C: <2.2 μg.kg^{-1}; griseofulvin: <1.2 μg.kg^{-1}; mycophenolic acid:2.5 μg.kg^{-1}), and two with one mycotoxin each (PHCC 4, mycophenolic acid: 4.28 μg.kg^{-1} ; PHCC8, sterigmatocystin: 3.80 μg.kg^{-1}).	Our results emphasize the need to implement corrective measures to avoid the mycotoxins contamination, and highlight the need for further studies addressing mycotoxins in clinical environments.	[9]
	Impinger air samples (*n* = 41) and HVAC filter samples (*n* = 12)	patulin, nivalenol, deoxynivalenol-3-glucoside, deoxynivalenol, fusarenon-X, α-zearalanol, β-zearalanol, β-zearalenol, α-zearalenol, zearalanone, zearalenone, T-2 tetraol, deepoxydeoxynivalenol, neosolaniol, 15-acetyldeoxynivalenol, 3-acetyldeoxynivalenol, monoacetoxyscirpenol, diacetoxyscirpenol, aflatoxin M1, aflatoxin B1, aflatoxin B2, aflatoxin	Mycotoxins were detected both in air and HVAC filter samples. Nine air samples were contaminated (ng/mL) with 1–5 different mycotoxins in the same sample. The mycotoxins detected were fumonisins B1 (2	Detection of mycotoxins in both types of samples (air and HVAC filters) reinforces the relevance of studying mycotoxins presence in clinical environment.	[10]

		G1, aflatoxin G2, fumonisin B1, fumonisin B2, fumonisin B3, T-2 triol, roquefortine C, sterigmatocystin, griseofulvin, T-2 toxin, HT-2 toxin, ochratoxin A, ochratoxin B, mycophenolic acid, mevinolin.	samples, < 4.3), B2 (6 samples, < 2.8–8.8) and B3 (1 sample, < 3.9), roquefortine C (1 sample, < 0.7) and ochratoxin A (9 samples, < 0.6–2.25) and ochratoxin B (1 sample, < 0.8), being ochratoxin A the most prevalent and fumonisin B2 the mycotoxin with the highest measured values. Concerning HVAC filters, four samples were contaminated (ng/g) with 1 and 2 mycotoxins in the same filter. The mycotoxins detected were fumonisin B2 (3 samples, < 7 0.6–21.4), ochratoxin A (1 sample, 6.70), mycophenolic acid (1 sample, 40.3) and sterigmatocystin (1 sample, < 2.9). Also in HVAC filters, fumonisin B2 was the most prevalent mycotoxin, exhibiting highest measured values.		
Dairies	Cattle feed (*n* = 9): feed available for lactating cows and maternity (*n* = 2), raw materials normally used to prepare the animals' feed (*n* = 1); expanded soybean and minerals (*n* = 1); grasses (*n* = 1); liquid cane molasses (*n* = 1); corn sealing (*n* = 1); brewers' grain (*n* = 1), and bagasse soybeans (*n* = 1), litter from the maternity sector (*n* = 1).	Trichothecenes, ZEA, and fumonisins, aflatoxins and Ochratoxin A	From the 16 mycotoxins analyzed, only AFB2, AFG1, and AFG2 were not detected in the samples. Regarding the mycotoxins detected, ZEA was detected in all the samples (0.6–155 ng g^{-1}) with the highest value in the litter sample. Deoxynivalenol was reported in 8 of the 10 samples (<3–197 ng g^{-1}). Ochratoxin A was detected in five samples (<0.4 to 4.53 ng g^{-1}). T-2 (<0.6–2.95 ng g^{-1})	The results point to the possible contamination of milk by several mycotoxins and raise the possibility of occupational exposure to mycotoxins due to feed contamination	[11]

			and HT-2 (<2–19.6 ng g^{-1}) were detected in four of the same samples. NIV (<3–87.1 ng g^{-1}), FB1 (<5–873 ng g^{-1}) and FB2 (<5–292 ng g^{-1}) were detected in three samples; FB3 was detected in two samples (10.6 and 94.7 ng g^{-1}), and 3-AcDON (3.5 ng g^{-1}), MAS (2.9 ng g^{-1}), and DAS (1.45 ng g^{-1}) were only reported once.		
Bakeries	Air samples (*n* = 53) and settled dust samples (*n* = 11)	patulin, nivalenol, deoxynivalenol-3-lucoside, deoxynivalenol, fusarenon-X, α-zearalanol, β-zearalanol, β-zearalenol, α-zearalenol, zearalanone, zearalenone, T2 tetraol, deepoxydeoxynivalenol, neosolaniol, 15-acetyldeoxynivalenol, 3-acetyldeoxynivalenol, monoacetoxyscirpenol, diacetoxyscirpenol, aflatoxin M1, aflatoxin B1, aflatoxin B2, aflatoxin G1, aflatoxin G2, fumonisin B1, fumonisin B2,fumonisin B3, T2 triol, roquefortine C, griseofulvin, T2 toxin, HT2 toxin, ochratoxin A, ochratoxin B, mycophenolic acid, mevinolin	None of the 36 mycotoxins were detected in air samples. Regarding settled dust, all samples showed contamination with 6 to 8 mycotoxins in each sample. DON was clearly the mycotoxin measured in higher amounts as all the samples identified quantifiable results.	The information regarding settled dust contamination by several mycotoxins was useful for the awareness for the presence of this occupational risk and to ponder the raw material (e.g., flour) as an indoor contamination source.	[12]
Fresh Bread Dough	HBM (urine) Workers (*n* = 21) and controls (*n* = 19) and settled dust) samples (*n* = 1)	Urine analyses (aflatoxins B1/2/G1/2/m1, alternariol, alternariol-monomethyletherand altenuene).<br>Settled dust (patulin, nivalenol, deoxynivalenol-3-glucoside, deoxynivalenol, usarenon-X, deepoxy-deoxynivalenol, α-zearalanol, β-zearalanol, β-zearalenol, α-zearalenol, zearalenone, T-2 toxin, T-2 tetraol, T-2 triol, neosolaniol,<br>15-acetyldeoxynivalenol, 3-acetyldeoxynivalenol, monoacetoxyscirpenol, diacetoxyscirpenol, aflatoxin M1, aflatoxin B1, aflatoxin B2, aflatoxin G1, aflatoxin G2, fumonisin B1, fumonisin B2, fumonisin B3, roquefortine C, griseofulvin, HT-2 toxin, ochratoxin A, ochratoxin B, mycophenolic acid, as well as mevinolin.	DON-GlcA was the most prominent biomarker found in both groups but at the highest levels in the samples from the workers' group. AFM1 showed lower concentrations compared to DON-GlcA but also only measured in the workers group. OTA was detected in both groups showing that 58% (23/40) of all the individuals enrolled in the study were exposed. CIT was measured in only one sample from the control group.<br>None of the 36 mycotoxins were detected in air samples. Regarding settled	The workers group, due to their high contact with flour dust, revealed a higher exposure to DON.	[13]

			dust, all samples showed contamination with 6 to 8 mycotoxins in each sample. DON was clearly the mycotoxin measured in higher amounts as all the samples identified quantifiable results.		
One Central Hospital Lisbon	Electrostatic dust cloths (*n* = 16)	15-Acetyldeoxynivalenol, 3-Acetyldeoxynivalenol, Aflatoxin B1,Aflatoxin B2 Aflatoxin G1,Aflatoxin G2 Aflatoxin M1,α-Zearalanol α-Zearalenol,β-Zearalanol β-Zearalenol,Deepoxydeoxynivalenol,Deoxynivalenol Diacetoxyscirpenol, DON-3-Glucosid,Fumonisin B1 Fumonisin B2,Fumonisin B3, Fusarenon-X,Gliotoxin Griseofulvin,HT-2 Toxin,Mevinolin,Moniliformin,Monoacetoxyscirpenol,Mycophenolic acid,Neosolaniol,Nivalenol Ochratoxin A,Ochratoxin B Patulin,Roquefortine C Sterigmatocystin,T-2 Tetraol,T-2 Toxin,T-2 Triol Zearalanone,Zearalenon	There were no mycotoxins detected.	This study supports the importance of considering exposure to complex mixtures in indoor environments.	[14]
One Central Hospital - Oporto	Impinger air samples (*n* = 15) and HVAC filter samples (*n* = 2)	15-Acetyldeoxynivalenol, 3-Acetyldeoxynivalenol,Aflatoxin B1,Aflatoxin B2,Aflatoxin G1,Aflatoxin G2,Aflatoxin M1,Deepoxy-deoxynivalenol,Deoxynivalenol,Deoxynivalenol-3-glucoside,Diacetoxyscirpenol,Fumonisin B1,Fumonisin B2,Fumonisin B3,Fusarenon X,Griseofulvin,HT-2 toxin Mevinolin,Monoacetoxyscirpenol,Mycophenolic acid, Neosolaniol,Nivalenol, Ochratoxin A,Ochratoxin B Patulin,Roquefortine C,Sterigmatocystin,T-2 tetraol,T-2 toxin,T-2 triol Zearalanone,Zearalenone α-Zearalanol,α-Zearalenol β-Zearalanol,β-Zearalenol	There were no mycotoxins detected.	This study supports the importance of considering exposure to complex mixtures in indoor environments.	[15]

Human biomonitoring (HBM); Aflatoxin B1 (AFB1); Zearalenone (ZEA); deoxynivalenol (DON); ochratoxin A (OTA); Aflatoxin M1 (AFM1); deoxynivalenol-glucuronide (DON-GlcA); Enniatin B (EnB); Citrinin (CIT); dihydrocitrinone (DH-CIT); electrostatic dust collector (EDC); filtering respiratory protective devices (FRPD).

References

1. Viegas, S.; Veiga, L.; Figueiredo, P.; Almeida, A.; Carolino, E.; Sabino, R.; Veríssimo, C.; Viegas, C. Occupational exposure to aflatoxin B1 in swine production and possible contamination sources. *J. Toxicol. Environ. Health Part A* **2013**, *76*, 944–951.
2. Viegas, S.; Assunção, R.; Martins, C.; Nunes, C.; Osteresch, B.; Twarużek, M.; Kosicki, R.; Grajewski, J.; Ribeiro, E.; Viegas, C. Occupational exposure to mycotoxins in swine production: Environmental and biological monitoring approaches for exposure assessment. *Toxins* **2019**, *11*, 78.
3. Viegas, S.; Veiga, L.; Malta-Vacas, J.; Sabino, R.; Figueiredo, P.; Almeida, A.; Viegas, C.; Carolino, E. Occupational exposure to aflatoxin (afb1) in poultry production. *J. Toxicol. Environ. Health Part A* **2012**, *75*, 1330–1340.
4. Viegas, S.; Veiga, L.; Almeida, A.; dos Santos, M.; Carolino, E.; Viegas, C. Occupational Exposure to Aflatoxin B1 in a Portuguese Poultry Slaughterhouse. *Ann. Occup. Hyg.* **2015**, *60*, 176–183.
5. Viegas, C.; Faria, T.; Cebola de Oliveira, A.; Aranha Caetano, L.; Carolino, E.; Quintal-Gomes, A.; Twarużek, M.; Kosicki, R.; Soszczyńska, E.; Viegas, S. A new approach to assess fungal contamination and mycotoxins occupational exposure in forklifts drivers from waste sorting. Mycotoxin Res. **2017**, *33*, 285–295.
6. Viegas, C.; Dias, M.; Almeida, B.; Aranha Caetano, L.; Carolino, E.; Quintal Gomes, A.; Twaruzek, M.; Kosicki, R.; Grajewski, J.; Marchand, G.; et al. Are workers from waste sorting industry really protected by wearing Filtering Respiratory Protective Devices? The gap between the myth and reality. *Waste Manag.* **2020**, *102*, 856–867.
7. Viegas, C.; Twarużek, M.; Dias, M.; Almeida, B.; Carolino, E.; Kosicki, R.; Soszczyńska, E.; Grajewski, J.; Aranha Caetano, L.; Viegas, S. Assessment of the microbial contamination of mechanical protection gloves used on waste sorting industry: A contribution for the risk characterization. Environ. Res. **2020**, *189*, 109881.
8. Viegas, S.; Veiga, L.; Figueiredo, P.; Almeida, A.; Carolino, E.; Viegas, C. Assessment of Workers' Exposure to Aflatoxin B1 in a Portuguese Waste Industry. *Annals of Occupational Hygiene* **2014**, *59*(2), 173–181. doi:10.1093/annhyg/meu082.
9. Viegas, C.; Almeida, B.; Monteiro, A.; Paciência, I.; Cavaleiro, J.R.; Carolino, E.; Quintal-Gomes, A.; Twarużek, M.; Kosicki, R.; Marchand, G.; et al. Settled dust assessment in clinical environment: Useful for the evaluation of a wider bioburden spectrum. *Int. J. Environ. Health Res.* **2019**, *26*, 1–19.
10. Viegas, C.; Almeida, B.; Monteiro, A.; Aranha Caetano, L.; Carolino, E.; Quintal-Gomes, A.; Twarużek, M.; Kosicki, R.; Marchand, G.; Viegas, S. Bioburden in healthcare centers: Is the compliance with Portuguese legislation enough to prevent and control infection? *Build. Environ.* **2019**, *160*, 106226.
11. Viegas, S.; Assunção, R.; Twarużek, M.; Kosicki, R.; Grajewski, J.; Viegas, C. Mycotoxins feed contamination in a dairy farm—Potential implications for milk contamination and workers' exposure in a One Health approach. *J. Sci. Food Agric.* **2019**, doi:10.1002/jsfa.10120.
12. Viegas, C.; Faria, T.; Aranha Caetano, L.; Carolino, E.; Quintal-Gomes, A.; Twaruzek, M.; Kosicki, R.; Viegas, S. Characterization of Occupational Exposure To Fungal Burden in Portuguese Bakeries. *Microorganisms* **2019**, *7*, 234.
13. Viegas, S.; Assunção, R.; Nunes, C.; Osteresch, B.; Twaruzek, M.; Kosicki, R.; Grajewski, J.; Martins, C.; Alvito, P.; Almeida, A.; et al. Exposure Assessment to Mycotoxins in a Portuguese Fresh Bread Dough Company by Using a Multi-Biomarker Approach. *Toxins* **2018**, *10*, 342.
14. Viegas, C.; Twarużek, M.; Lourenço, R.; Dias, M.; Almeida, B.; Caetano, L.A.; Carolino, E.; Gomes, A.Q.; Kosicki, R.; Soszczyńska, E.; Viegas, S. Bioburden Assessment by Passive Methods on a Clinical Pathology Service in One Central Hospital from Lisbon: What Can it Tell Us Regarding Patients and Staff Exposure? *Atmosphere*. **2020**, *11*, 351.
15. Viegas, C.; Almeida, B.; Monteiro, A.; Paciência, I.; Rufo, J.; Aguiar, L.; Lage, B.; Diogo Gonçalves, L. M.; Caetano, L. A.; Carolino, E.; Gomes, A. Q.; Twarużek, M.; Kosicki, R.; Grajewski, J.; Teixeira, J. P.; Viegas, S.; Pereira, C. Exposure assessment in one central hospital: A multi-approach protocol to achieve an accurate risk characterization. *Environmental research*. **2020**, *181*, 108947.
